# Metabolic control of epigenetic rearrangements in B cell pathophysiology

**DOI:** 10.1098/rsob.220038

**Published:** 2022-05-18

**Authors:** Beatrice Calciolari, Greta Scarpinello, Laura Quotti Tubi, Francesco Piazza, Alessandro Carrer

**Affiliations:** ^1^ Department of Biology (DiBio), of the University of Padova, Padova, Italy; ^2^ Department of Medicine (DIMED), Hematology and Clinical Immunology Section, of the University of Padova, Padova, Italy; ^3^ Department of Surgical, Oncological and Gastroenterological Sciences (DiSCOG), of the University of Padova, Padova, Italy; ^4^ Veneto Institute of Molecular Medicine (VIMM), Padova, Italy

**Keywords:** B cell, metabolism, epigenetics, lymphoma

## Abstract

Both epigenetic and metabolic reprogramming guide lymphocyte differentiation and can be linked, in that metabolic inputs can be integrated into the epigenome to inform cell fate decisions. This framework has been thoroughly investigated in several pathophysiological contexts, including haematopoietic cell differentiation. In fact, metabolite availability dictates chromatin architecture and lymphocyte specification, a multi-step process where haematopoietic stem cells become terminally differentiated lymphocytes (effector or memory) to mount the adaptive immune response. B and T cell precursors reprogram their cellular metabolism across developmental stages, not only to meet ever-changing energetic demands but to impose chromatin accessibility and regulate the function of master transcription factors. Metabolic control of the epigenome has been extensively investigated in T lymphocytes, but how this impacts type-B life cycle remains poorly appreciated. This assay will review our current understanding of the connection between cell metabolism and epigenetics at crucial steps of B cell maturation and how its dysregulation contributes to malignant transformation.

## Graphical abstract

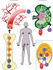

## 1. Introduction

B lymphocytes are central players in the immune response as terminally differentiated plasma B cells specialize in synthesizing and secreting large amounts of antibodies [1,2]. Here, we will review evidence showing metabolic control of the epigenome during B cell maturation.

Humoral immunity must be finely regulated to mount a rapid and efficient antibody response while containing adverse effects due to uncontrolled immune reaction or targeting of self-antigens [2]. This is achieved through a multistep process that is coordinated by stage-specific cytokines and transcription factors (TFs), which are critical to support the commitment of haematopoietic stem cells (HSC) into the B cell lineage and then the efficient maturation of pro-B cells into functional immune effectors [1,3,4]. Alterations of the cytokine-receptor circuitry can also drive oncogenesis, fostering cell proliferation or blocking terminal differentiation [5]. B cell-specific signalling pathways trigger the simultaneous expression and repression of hundreds of genes that determine cell identity, phenotype and function, all of which change considerably during the specification of B cell precursors [1,4]. Broad-ranging changes in gene expression profiles can be mediated by epigenetic remodellers that modify chromatin architecture to amplify (or restrain) the activity of master transcription factors [6,7]. In line with this concept, several epigenetic remodelling factors are differentially expressed throughout B cell specification [7,8] and gene targeting experiments in mice demonstrate their causative involvement (summarized in box 1).

Box 1. **Dissecting B-cell epigenetic reprogramming in mouse.**

**DNA methylation**

DNA can be methylated at cytosines in CpG motifs, enabling stable annotation of the genome usually associated with gene repression. Global levels of DNA methylation progressively decrease upon induction of B cell differentiation, with PCs and memory B cells having an extensively demethylated genome.

*Writers.* DNA methyltransferase (DNMT)-1/2/3a/3b. B cell-specific DNMT3a/b deletion (CD19-Cre;Dnmt3a/b^flox^) augments PC differentiation in mice, although it is reported to be largely dispensable for B cell specification [9]. However, DNMT3-deficient mice show GC hyperplasia after immunization [9].

*Erasers.* Ten-eleven translocation protein (TET)-1/2/3. Active DNA demethylation is critical at multiple steps of B cell specification. Indeed, genetic deletion of TET-2/3 in experimental mice causes impairments at early stages (pro-B cells, class switch recombination), GC expansion and terminal differentiation [10–12].

**Histone methylation**

Histone methylation has a variegated effect on gene transcription: it can induce either activation or repression, or establish a poised chromatin state (facultative heterochromatin) for more dynamic transcriptional regulation. Histone methyltransferases often form complexes and reciprocally influence their activity. As such, the role of histone methylation in B cell development is convoluted and still under examination.

*Writers.* Both lysine and arginine residues of histone proteins can be methylated by lysine (K) methyl transferases (KMTs) and peptidyl-arginine (R) methyl transferases (PRMT), respectively. Lysines can be attached with a different number of methyl moieties, defining the methylation state: mono-methylation (me1), di-methylation (me2) and tri-methylation (me3). In line, the list of annotated histone methyltransferases is long despite just a few stand out for their contribution to B cell specification.

Histone H3 is heavily methylated at lysine 4, 9, 27 and 36 that, when modified, significantly impact gene expression, chromatin organization and cell identity. Enhancer of Zeste Homolog 2 (EZH2) methylates H3K27, is significantly upregulated in GCs and coordinates the reset of H3K27 tri-methylation (erased genome-wide and gained at key loci) [13]. Indeed, EZH2-deficient mice fail to arrange the GC reaction. A similar effect is observed upon deficiency of the H3K79 methylatransferase DOT1 L, which also interacts with EZH2 [14].

By contrast, deletion of the H4K4 methyltransferase, lysine methyl transferase 2D (KMD2D or MLL2), determines an increase of GC B cells [15] due to epigenetic changes in pre-B cells. Interestingly, H3K9 methylation might be critical for class switch recombination: mice lacking the KMT G9a exhibit genome-wide reduction of H3K9me2 and decreased usage of Ig light chain despite modest effects on humoral immunity overall [16]. Similarly, mice deficient for the H3K9me3 methyltransferase Suv39h1 show impairment specifically in the expression of class A Ig [17].

*Erasers.* Peptidylarginine deiminase 4 (PADI4) converts methyl-arginine in citrulline, while two different classes of enzymes are responsible for histone de-methylation: FAD-dependent amine oxidase LSD1/2 and Jumonji C-containing histone demethylases (JHDM), which use free iron and a-KG as cofactors.

LSD1-deficient mice fail to mount an efficient humoral response. In fact, LSD1 supports differentiation of naive B cells into plasmablasts [18] and establishment of the GC reaction [19]. Lack of LSD1 maintains chromatin highly accessible in naive B cells and impair the repression of BLIMP-1- and BCL6-regulated genes.

**Histone acetylation**

Acetylation of histone proteins diminishes their positive charge, enhancing chromatin accessibility for transcription factors. Histone tails (particularly histone H4) can be heavily acetylated at discrete loci throughout the genome (gene promoters, enhancers, super-enhancers). Acetylated residues can also be recognized by bromodomain (BRD)-containing transcriptional activators. For these reasons, histone acetylation is invariably associated with active transcription.

*Writers.* A variegated class of histone acetyl transferases (HATs) have been annotated, with largely promiscuous activities. However, gene targeting experiments suggest some enzyme-specific functions.

B cell-specific (CD19-Cre) deletion of either *Ep300* or *Cbp*, two prominent and highly interconnected HATs, has negligible effects on humoral immunity although absence of p300 selectively impairs the count of pro-B cells. However, simultaneous ablation of the two enzymes dramatically impacts peripheral B cells number (transitional B cells and PCs) [20]. On the other hand, double-deficient mice lacking both *Gcn5* and *Pcaf* accumulate pro-B cells and fail to undergo efficient class switch recombination [21].

*Erasers.* Histone de-acetylase complexes (HDACs), along with NAD-dependent sirtuins (SIRTs), are responsible for the removal of acetyl groups from histones, liberating acetate. These two classes are composed by several members, often with redundant functions. HDAC involvement in B cell physiology and pathology is well documented. Indeed, HDAC4/5/7 form a large complex with master GC regulator, BCL6 and are essential for its repressor function.

Gene ablation in mice revealed that HDAC1 and HDAC2 are critical for pro-B cell proliferation while dispensable for mature B cell survival. Yet, HDAC1/2-null B cells are insensitive to IL4 stimulation [22]. By contrast, HDAC6 deletion in B cells has no obvious consequence [23].

Cell metabolism influences cell fate decisions through multiple mechanisms [24–26]; most notably, metabolite availability impacts the epigenome [27,28]. Numerous metabolic intermediates serve as substrates or cofactors for chromatin remodelling enzymes and their intracellular levels can be limiting for epigenetic reactions [29,30]. This empowers the cells with a potent tool to sense and quickly react to external stimuli, thus informing cell differentiation. The same machinery can be hijacked by oncogenes to drive tumorigenesis [31], and several metabolic checkpoints also exist to limit epigenomic instability and restrain B cell development [24]. This review focuses on the link between metabolic and epigenetic reprogramming in B cell maturation and lymphomagenesis. We put forward the idea that signalling and metabolic cues cooperatively contribute to the epigenetic remodelling of B cell precursors during their multi-step specification.

## 2. B cell development is a multistep process

**Figure 1.**
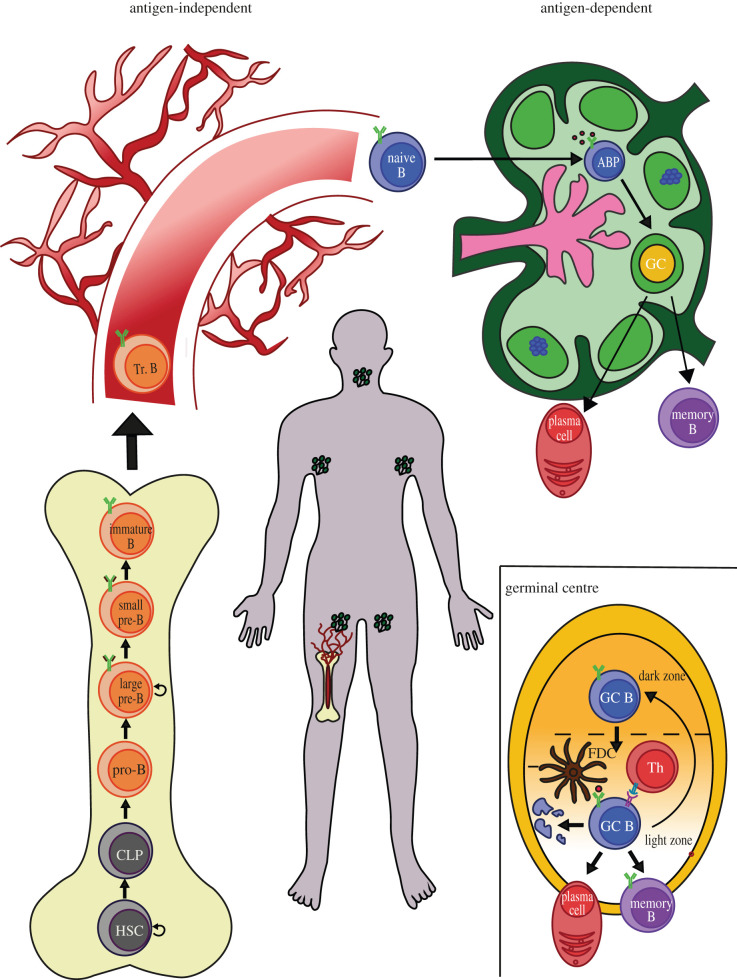
Multi-step B cell specification. The figure illustrates step-wise evolution of haematopoietic precursors (haematopoietic stem cells; HSCs) into terminally differentiated B cells—either plasma cells (PCs) or memory B cells (B_mem_). Cellular identity is progressively reshaped through a number of intermediary phenotypic and functional states, each characterized by unique marker expression. B cell specification begins in the bone marrow (bottom left corner), and immature B cells are eventually released into the circulation (top left corner). Upon antigen recognition, naive B cells seed into secondary lymphoid organs like the lymph nodes (top right corner). Maturation is finalized within germinal centres (GC), which are transient and highly heterogeneous histological structures highlighted in the blown out (bottom right corner). In the middle, representative human body and location of highlighted structures. Other abbreviations: CLP (common lymphoid precursor); Tr.B (transitional B cells); ABP (activated B cell precursors); GC B (germinal centre B cells); Th (T helper lymphocytes); FDC (follicular dendritic cells).

Lymphocytes are the effectors of adaptive immunity against infections [1]. Type B lymphocytes mount and execute a sophisticated immune response in that pathogens (and other stimuli) instruct B cell precursors to differentiate into cells that produce specific antigen-recognizing antibodies for optimal protection. B cells progressively specify their function starting from naive pro-B cells that germinate from haematopoietic stem cells by asymmetric cell division, through the intermediate stage of common lymphoid precursor (CLP; see below). A functional B cell receptor (BCR) is invariably expressed on their surface and is activated upon antigen encounter. This event initiates differentiation of naive B cells and ultimately leads to the generation of either antibody-secreting plasma cells or memory B cells (figure 1). A significant body of work has defined the transcriptional profiles of stage-restricted B cell populations [32–34].

The massive reprogramming of molecular and functional traits during B cell differentiation manifests epigenetic control [7,8]. The regulation of chromatin architecture and accessibility allows synchronous change of thousands of genes and brisk remodelling of cell identity. Indeed, compelling data show that stepwise expression/activation of several chromatin remodelling enzymes is necessary for the correct development of a functional B cell (box 1). In addition, B cells need to adapt their cellular metabolism to satisfy different energetic or anabolic needs, and thrive in different environments [35,36]. Metabolic sensing is indeed critical to proper B cell development and its dysregulation is associated with immunodeficiencies, autoimmune diseases and cancer [37,38]. Relatively less is known on whether and how epigenetic and metabolic reprogramming are reciprocally regulated, and how this connection is hijacked in B cell malignant transformation.

In mammals, B cells development starts in fetal liver and adult bone marrow (antigen-independent phase) and it is then completed in secondary lymphoid organs (antigen-dependent phase). Haematopoietic stem cells first commit to the lymphoid lineage and become common lymphoid progenitors (CLP). Next, CLP gradually mature to naive B cells through an ordered sequence of specification stages. The main event in this phase is the sequential rearrangement of immunoglobulin gene segments to assemble the IgH heavy (IgH) chains that are expressed on the surface of pre-B cells. IgHs associate with surrogate immunoglobulin light chains (IgL) and Igα-β signalling molecules to form the pre-BCR receptor. Signalling through a functional pre-BCR receptor induces recombination of the IgL genes. Pairing of IgH and IgL yields a full-fledged BCR receptor, which is expressed on the surface of immature B cells and known as IgM. At this point, immature B cells exit the bone marrow to disseminate in the bloodstream and become transitional B cells. While migrating to secondary lymphoid organs, transitional B cells express IgD on their cell surface and are negatively selected for autoreactivity [4,37,39,40].

**Figure 2.**
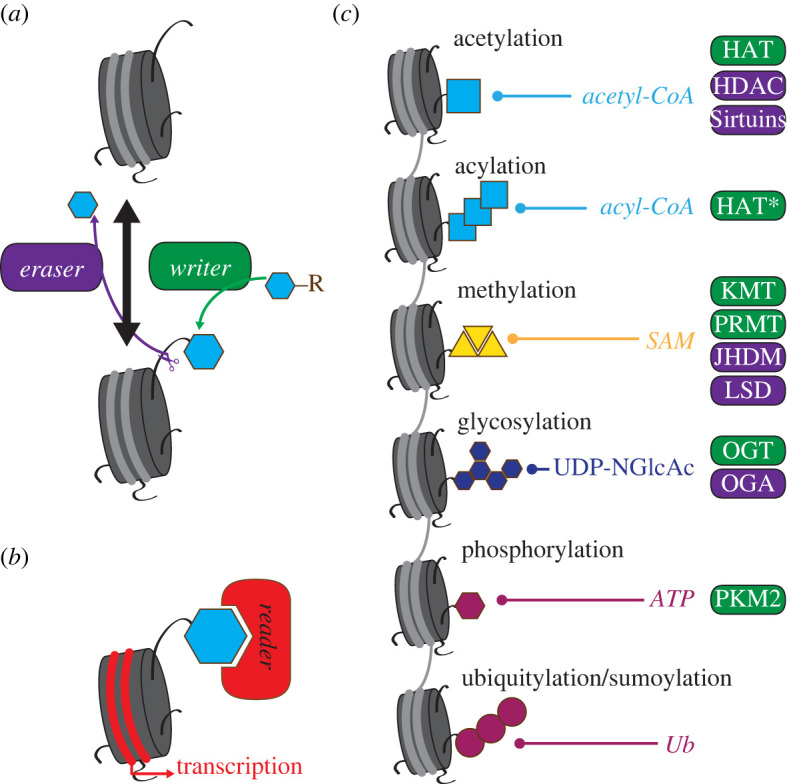
Histone modification by epigenetic enzymes. (*a*) Deposition and removal of reactive moieties (light blue) onto histone proteins by epigenetic *writers* and *erasers* respectively. Histones can switch between modified (bottom) and non-modified (top) states. (*b*) Epigenetic readers recognize specific histone modifications to regulate gene transcription in proximity. (*c*) Several types of histone modifications have been described. Most are linked to metabolites. For each modification, known classes of writers (green) and erasers (purple) are annotated. HAT (histone acetyl transferases; * denotes promiscuous activity); HDAC (histone deacetylase); KMT (lysine methyl transferase); PRMT (protein arginine methyl transferase); JMJC (Jumonji C-containing histone demethylase); LSD (lysine-specific histone demethylase); OGT (O-GlcNAc transferase); OGA (protein O-GlcNAcase); PKM2 (pyruvate kinase isoform M2); Ub (ubiquitin).

During the peripheral phase of B cell development, a specific antibody repertoire is built against a cognate antigen. This implies both iterative increase of BCR affinity through somatic hypermutation (SHM) and the generation of different classes of antibodies through class-switched recombination (CSR) [2]. Both processes rely on the activity of activation-induced cytidine deaminase (AID), which predisposes to DNA mutations, insertions and deletions. During CSR, which occurs in early activated B cells, AID targets the constant region of Ig loci, while its activity is retargeted to variable region Ig loci during SHM [40]. At the same time, these mechanisms intrinsically predispose to cancer in that both CSR and SHM can generate genetic alterations associated with B cell lymphomas [41] (figure 2). As well as AID-mediated DNA editing, epigenetic aberrations often facilitate hypermutant phenotypes and lymphomagenesis [42].

Activation of mature naive B cells in the periphery requires two signals: the first signal is generated upon antigen binding to the BCR receptor; a second signal can be provided by T-helper lymphocytes [4]. Upon low-affinity antigen recognition, activated B cells precursors (ABPs) seed the germinal centre reaction [40,43]. Germinal centres (GCs) are transient dynamic structures in lymphoid organs (mostly lymph nodes) where BCR affinity is improved through iterative rounds of somatic hypermutations (SHM) of the Ig genes [40]. Positively selected cells then cycle between the dark zone (DZ), where B cells proliferate and undergo SHM, and the light zone (LZ) where B cells are screened for their BCR affinity until they terminally differentiate into plasma cells (PC) or memory B cells (B_mem_) [40,43,44]. On the other hand, negatively selected ABPs undergo apoptotic cell death. Recent molecular analyses have revealed a more convoluted organization of GC topology and possibly the existence of a grey zone (GZ) where clonal expansion might be confined to and spatially separated from the DZ area where cells strictly undergo SHM [45]. In all, GCs are transient anatomical sites where mature B cells improve their affinity for their antigen and expand high-responding clones, before committing to terminal differentiation (figure 1).

Clearly, GCs host a tightly regulated reaction where cells are maintained in a mature yet undifferentiated state and transiently manifest a number of traits that are common to or overplayed in cancer cells: accelerated proliferation and clonal expansion, genome instability, resistance to DNA damage, immune evasion and terminal differentiation blockade [5,38]. These features are coordinated at the transcriptional level by transient expression of *B-Cell Lymphoma protein 6* (BCL6), a zinc-finger transcription factor that mediates the cellular response downstream of BCR/IL4 stimulation [46]. BCL6 expression is exclusive to GC B cells and finely regulated at the epigenetic level, as discussed later.

BCL6 is a transcriptional repressor [46]. However, by suppressing microRNA-155 and microRNA-361, it eventually upregulates *AICDA* [47], the gene encoding for *Activation-induced cytidine deaminase* (AID). AID is an endogenous DNA mutator that mediates SHM at the Ig locus, where its activity is preferentially confined [41]. Epigenetic mechanisms constrain both AICDA expression and chromatin accessibility, as discussed below. BCL6 and AID are selective markers of GC B cells [40]. The BCL6-orchestrated transcriptional program that establishes the transient GC reaction is subsequently terminated by *B Lymphocyte-Induced Maturation Protein -1* (BLIMP-1), which in turn drives the exit from GC reaction cycling and coordinates plasma cell differentiation [48]. In line, BLIMP-1 is a well-characterized PC marker [49]. Factors involved in B_mem_ induction are less characterized [43,49].

## 3. Epigenetic reprogramming during B cell maturation

Within the nucleus of eukaryotic cells, DNA is folded into a compact, hierarchically organized structure known as chromatin. The modular unit of nuclear chromatin is called a nucleosome and consists of DNA wrapped around a core of highly conserved histone proteins. Chromatin is a highly plastic structure that dynamically changes during the cell cycle and across phenotypic states [50,51].

Histone or DNA post-translational modifications (methylation and acetylation being the most abundant) dictate chromatin architecture and accessibility; at the same time, they represent epigenetic marks that modulate the binding of transcriptional activators or repressors to regulate wide-range gene expression. Chromatin marks are set by a group of enzymes with the ability to attach, remove or modify covalent bonds of histone tails and nucleotide residues that are then recognized by dedicated transcriptional regulators. These proteins can accordingly be classified into *writers*, *erasers* and *readers* (figure 2). By defining the chromatin landscape of the cell, epigenetic modifications control developmental and postnatal lineage specification of immature cells, as well as plasticity of terminally differentiated cells [51–53].

Differentiation of immune cells is initiated by stage-specific transcription factors [34]. TFs recruitment to target genes is not sufficient *per se*, but must be either directed or followed by remodelling of the chromatin landscape. This is key to the proper function of differentiation-promoting TFs for several reasons; most notably, (a) to constrain transcriptional activation/repression to a specific set of genes, (b) to coordinate gene transcription in time, (c) to maintain lineage identity upon differentiation, and (d) preserve long-lived and heritable memory of activation.

Compelling evidence links epigenetic reprogramming to B cell differentiation, particularly during the transient formation of GCs, and the subsequent mounting of a proper immune response [52–54]. Genetic studies in mice have highlighted the role of epigenetic remodelling enzymes in B cell development showing that ablation of epigenetic players results in impaired or halted B cell maturation with varying degrees of immunodeficiency. Results from these studies have been extensively reviewed elsewhere [7,38,42] and are summarized here in box 1. Interestingly, ablation of counterbalancing enzymes can result in similar phenotypes (i.e. B cell-specific deficiency), as either DNA-methylating *Dnmt3b* or DNA-demethylating *Tet2* determines hyperplasia of germinal centre B cells [9,10], clearly indicating that epigenetic modifications are highly dynamic during differentiation. The GC reaction is initiated by IL4-driven upregulation of *BCL6*. Its expression is limited to GC B cells. Temporal- and lineage-specific restrictions are enforced through epigenetic mechanisms: both the proximal promoter and a distal enhancer are heavily modified by histone acetylation and methylation, which influence gene expression [55–57]. Moreover, BCL6 genomic locus constitutes a B-cell ‘super-enhancer’ (SE). SEs are large (hundreds of megabases) genomic elements characterized by superior histone acetylation that define cellular identity, by imposing chromatin topology [58]. For instance, AID targets genome territories created by B-cell SEs [41].

In humans, low B cell count or autoimmunity are observed in patients affected by rare genetic diseases caused by inactivating mutations at genes encoding for epigenetic remodellers (i.e. centromeric instability-facial anomalies, *DNMT3B*; and Kabuki syndrome type 1, *KMT2D*) [59,60]. Mutations are also abnormally frequent in B cell malignancies as revealed by multiple cohort studies [38]. This has fostered the testing of several epigenetic inhibitors for the treatment of immunological disorders, sometimes with notable success. The EZH2 inhibitor Tazemetostat (TAZVERIK, Epizyme, Inc) has been approved for relapsed or refractory follicular lymphoma (FL) by the FDA after accelerated review of a very successful single-arm clinical trial [61]. But despite being amenable to therapeutic attack, epigenetic remodelling has proven to be an elusive target in many instances due to the redundancy and promiscuity of most enzymes but also to the dynamic nature of epigenetic modifications. In addition, research now shows that metabolic factors affect the response to epigenetic therapies in tumours, including haematological cancers [62–65]. Metabolic intermediates can be manipulated by dietary or pharmaceutical interventions so this notion can pave the way to exploiting metabolism to enhance therapeutic success.

## 4. Metabolic reprogramming during B cell maturation

Cell metabolism is a well-characterized set of biochemical reactions responsible for the generation and storage of energetic equivalents, the maintenance of redox homeostasis, the synthesis of biologically active macromolecules and the processing of organic waste [66]. These tasks are executed through interchained reactions that break down carbon sources (catabolism) to obtain simpler intermediates that are subsequently used as building blocks for the synthesis of lipids, amino acids, sugars and nucleotides (anabolism) [67]. The cellular metabolic network is complex and highly dynamic to accommodate different needs or nutrient supply. It unfolds around the reactions that compose the tricarboxylic acid (TCA, also known as Krebs) cycle, which takes place in the mitochondria; the TCA cycle works as pivotal hub for cellular metabolism, with several intermediates being positioned at the crossroad of multiple metabolic pathways. The activity of mitochondria and the wiring of carbon atoms in and out the TCA cycle are regulated at multiple levels [67]. This metabolic flexibility allows the cell to adjust its redox status as metabolic reactions can either generate or consume reducing equivalents like NAD(H) and FAD(H) [67]. In simple terms, metabolism is a biochemical network that maintains life by processing carbon (and nitrogen) units from nutrients and adapting to different environments.

An underrated aspect of cell metabolism is its influence on signalling events, as the availability of certain metabolites can affect the stability or activity of several enzymes and, most notably for this review, the status of the epigenome [27,30]. At the same time, signalling cues regulate metabolic reactions [68,69]. This establishes a two-way relationship between signalling and metabolism that is often hijacked by oncogenes in order to sustain cell division (e.g. through the synthesis of DNA precursors and lipids) or promote identity shifts through the regulation of the epigenome.

**Figure 3.**
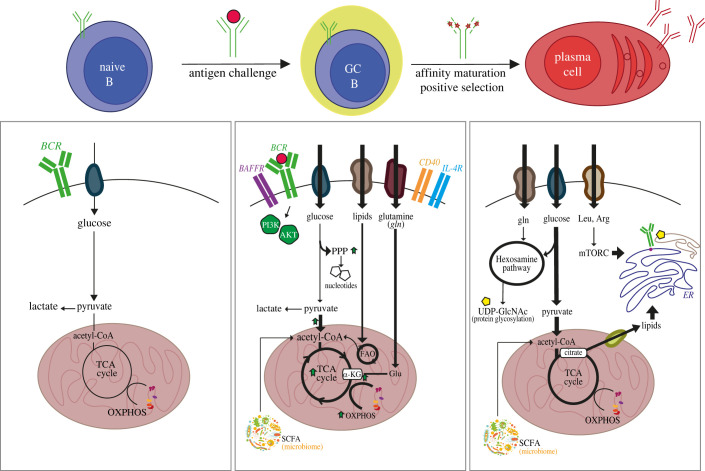
Metabolic reprogramming during B cell maturation. Rewiring of B cell metabolism between naive B cells, germinal centre B cells and plasma cells is illustrated. Cells can uptake a number of nutrients from the microenvironment, but at steady state they rely on glucose catabolism (left panel). Activation by antigen recognition triggers the uptake and catabolism of different carbon sources, which are then channelled into multiple pathways according to anabolic demands. Differentially used pathways are highlighted. Thicker lines denote enhanced flux. Elevated metabolite levels are denoted by green upward arrows. BCR (B cell receptor); TCA (tricarboxylic acid); OXPHOS (oxidative phosphorylation); FAO (fatty acid oxidation); PPP (pentose phosphate pathway); BAFFR (B cell activating factor receptor); Glu (glutamate); Leu (leucine); Arg (arginine); UDP-GlcNAc (UDP-glucose-N-acetylamine); SCFA (short chain fatty acid); ER (endoplasmic reticulum).

Cell metabolism is extensively reprogrammed across different stages of B cell specification [70], consequence of marked changes in the local microenvironment as cells migrate to secondary lymphoid organs, in energy demands as cells execute different functions, and in cytokine stimulations as cells receive different inputs from antigens and antigen-presenting cells. Stage-specific metabolic remodelling is pivotal for an effective humoral response. This has been reviewed elsewhere [35,36] and major findings to date are summarized in figure 3.

In brief, proliferative B cells (early BM B cells, activated B cells, GC B cells) exhibit high metabolic demands and profound rewiring of nutrient utilization, while their resting counterparts (naive B cells, memory B cells) display a more canonical metabolic status. During early developmental stages, pre-BCR signalling and IL-7 support large pre-B cells proliferative burst by reprogramming their metabolic profile toward anabolism [71]. After that, B cells turn to the immature naive stage and become quiescent. Naive B cells migrate to the spleen as transitional B cells, which are more metabolically active than their BM counterparts [72]. However, the turning event in B cell specification is antigen recognition, which marks a major switch in cell fate and also drives the most extensive metabolic rewiring. The B cell activation programme is initiated by cell-surface molecules including the BCR, CD40 (for T-dependent antigens), TLRs (for T-independent antigens) and IL-4R that elicit cell cycle progression, proliferation and anabolic growth. Activated B cells also undergo a balanced increase of both anaerobic glucose metabolism and oxidative phosphorylation, as confirmed by concomitant increase in lactate production, pyruvate oxidation and mitochondrial mass upon antigen challenge [57,73,74]. This vigorous metabolic activity is sustained by enhanced glucose, amino acid (i.e. glutamine) and lipid uptake in GC B cells that anaplerotically support mitochondrial respiration and nucleotide synthesis [73–76]. Finally, plasma cell differentiation poses distinct challenges as cells demand larger ER, sustained protein synthesis and glycosylation, together with an efficient secretory compartment. These demands are met through extensive rewiring of acetyl-CoA-derived intermediates, as discussed later. Major changes in metabolic flux are illustrated in figure 3.

A key metabolic regulator that promotes humoral immunity and controls several stages of B cell development is the *mammalian Target of Rapamycin Complex 1* (mTORC1) [77]. mTORC1 is a serine/threonine kinase which plays a pivotal role in eukaryotic cell metabolism, governing the balance between anabolism and catabolism. mTORC1 coordinates cell growth, survival and proliferation with the metabolic activity of the cell by sensing energy levels and environmental signals such as growth factors, nutrients and oxygen [78]. mTORC1 is essential during early B cell development and GC formation [79], while dysregulation of mTORC1 activity contributes to metabolic rewiring of cancer cells in haematological malignancies [80].

However, many of the metabolic features that characterize each stage of B cell development have not been defined yet and more work is necessary to understand the impact of metabolic reprogramming on B cell pathophysiology.

## 5. Metabolic control of the epigenome

**Figure 4.**
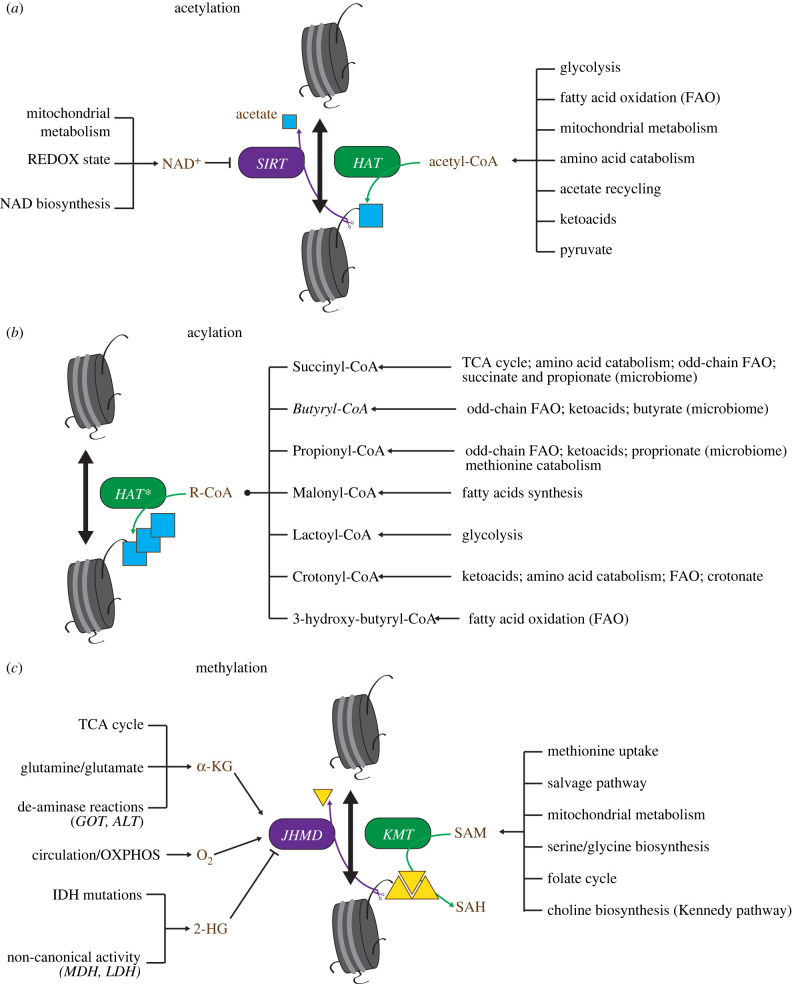
Metabolites impact the epigenome. Metabolites impact chromatin status serving as cofactor or substrates for enzymes that establish or remove epigenetic marks, including histone acetylation (*a*), acylation (*b*) and methylation (*c*). Chromatin-impacting metabolites are highlighted in red. Pathways that determine their intracellular availability are showed. In (*b*) R-CoA defines a number of acyl-CoA species listed on the right, along with major pathways by which they can be generated. HAT (histone acetyl transferases); SIRT (sirtuins); KMT (lysine methyl transferase); JHMD (Jumonji C-containing histone demethylase); NAD (nicotinamide adenine dinucleotide); a-KG (alpha-ketoglutarate); 2-HG (2-hydroxyglutarate); SAM (S-adenosylmethionine); SAH (S-adenosylcysteine).

Outside phosphorylation, covalent modification of histones requires metabolites as cofactors or donor substrates (figure 4); moieties for methylation and acetylation (the two most abundant modifications) are solely provided by S-adenosylmethionine (SAM) and acetyl-coenzyme A (acetyl-CoA), respectively [31]. As their availability fluctuates across developmental stages, metabolic dependencies of the epigenome might explain why changes in cellular metabolism are instrumental for multi-step B cell specification.

SAM is the universal methyl donor. It is the main product of methionine catabolism and an intermediate in multiple pathways, including one-carbon metabolism that generates methyl units from glycolysis (figure 4). SAM levels are maintained high in human stem cells, while their decrease poises cells for differentiation through the reprogramming of the epigenome at stemness-associated genes [81]. In addition, methionine availability constrains tumour cells proliferation and cancer stem cells plasticity [82,83].

Together with methylation, histone acetylation is the most significant epigenetic mark. It depends on the abundance of acetyl-CoA, which is a pivotal metabolite and the obligate acetyl donor for post-translational protein acetylation [84] (figure 4). Acetyl-CoA is the entry molecule of the TCA cycle and it is also required for several anabolic reactions (lipid synthesis, protein glycosylation, and others). Outside of mitochondria, acetyl-CoA is produced by ATP-citrate lyase (ACLY), acetate synthetase-2 (ACSS2) and nuclear-translocated pyruvate dehydrogenase (PDH), that metabolize different substrates and are preferentially localized in different sub-cellular compartments [85]. Compelling evidence demonstrates that acetyl-CoA levels are in equilibrium with global levels of histone acetylation under both physiological and pathological conditions [86–89]. For example, acetyl-CoA sustains elevated levels of histone acetylation in pluripotent stem cells while their commitment to differentiation reprograms central carbon metabolism and lowers acetyl-CoA abundance [88]. In T cells, increase of nucleo-cytoplasmic acetyl-CoA promotes differentiation and effector function inducing acetylation at the *Ifnγ* locus [90]. Several other acyl-CoA species have been shown to covalently bind histone proteins due to promiscuous activity of multiple HATs (figure 4); this is often competitive with histone acetylation [91], although the functional role of these modifications is not always clear. Propionyl-CoA is a low abundant metabolite that can derive from the catabolism of odd-chain fatty acids, ketoacids or the conjugation of microbiome-derived propionate [92]. Its availability is decreased during leukemic cell differentiation along with propionylation of lysine 23 on histone H3 [93]. Although this observation remains correlative it calls attention on less-represented histone modifications that may play a subtle but significant role in lymphocyte differentiation.

Removal of the methyl group from histones and DNA is performed by α-ketoglutarate (α-KG)-dependent dioxygenases, a relatively large family of oxidoreductase enzymes that utilizes α-KG and Fe^2+^ as cofactors and most notably includes the Jumonji histone demethylases and the TET hydroxylases [94]. Hence, levels of α-KG, which fluctuate in response to signalling and environmental cues, can influence the activity of DNA/histone demethylases and ultimately dictate cellular reprogramming. By contrast, fumarate, succinate and the oncometabolite (not detectable in normal cells) 2-D-hydroxyglutarate (2-D-HG) are competitive inhibitors of α-KG. The α-KG:succinate ratio is dynamic across developmental stages and affects methylation at loci critical for differentiation [95]. The relevance for tumorigenesis is epitomized by Isocitrate DeHydrogenase (IDH) mutations, which are common in brain tumours and acute myeloid leukaemia (AML) [96], and result in a neomorphic activity that causes the production of 2-D-HG. IDH mutations cause a hypermethylation phenotype that is responsible for cellular de-differentiation and hyperproliferation [97] (figure 4). These findings highlight an important level of regulation of the epigenome, where the abundance of critical metabolites fluctuates within a range that influences the activity of chromatin-remodelling enzymes.

Sirtuins represent another prominent class of chromatin erasers. These are histone deacetylases that are sensitive to NAD+ availability. Cellular redox status and nutrient utilization significantly impact the NAD+/NADH ratio and histone deacetylation through sirtuin activity [30]. Compartmentalization of NAD+ also influences sirtuin function in the nucleus [98].

In summary, cell differentiation is often controlled by metabolic sensing through the modification of the epigenetic landscape. Mounting evidence shows that nutrient availability and signalling events impact the levels of SAM, acetyl-CoA and α-KG, together with NAD+/NADH ratio. Fluctuations of these metabolites can be sensed by cells and integrated into the epigenome to inform cell fate decisions (e.g. differentiation, changes in cellular identity, phenotypic skewing). Of note, a number of key metabolic enzymes are also present in the nucleus, where they are recruited by specific stimuli, are enzymatically active and can bind to TFs or epigenetic regulators [92,99–102]; their nuclear compartmentalization is probably required for a quicker and more sensitive control of the epigenome [103].

## 6. Metabolic-dependent epigenetic reprogramming in multi-step B cell development

The rewiring of cellular metabolism, driven by both receptor signalling and nutrient availability, allows B cells to coordinate stage-specific metabolic needs with changing microenvironments [35] and it supports growth, proliferation but also phenotypic remodelling. Here, we will review how stage-specific changes in metabolite availability influence the epigenome and the developmental fate of B cells.

### 6.1. Naive B cells

Haematopoietic precursors exploit metabolic cues to maintain stemness traits and expand the pool of progenitors, or alternatively commit to B cell specification. Indeed, preference for carbon sources alternative to glucose (e.g. glutamine, fatty acids) can trigger asymmetric cell division and self-renewal [104,105]. Whether nutrient selection has consequences on chromatin remodelling remains somehow unclear.

Poor methionine dietary intake leads to low B cell count [106]; at the same time, reduced SAM abundance activates a cell cycle checkpoint while inhibition of SAM biosynthesis halts proliferation of non-tumorigenic FL5.12 pre-B cells [107]. Together this suggests that SAM-mediated high methylation potential might be necessary for naive B cell renewal. In line, elevated histone and DNA methylation are observed in early stages of B-cells development in the bone marrow [108]. However, an undisputed link between methionine metabolism, histone methylation and pre-B cell renewal has not been established yet.

*In vitro* studies have shown that the proliferation of FL5.12 cells is dependent on ACLY-mediated acetyl-CoA production [109,110]. Stimulation with IL-13 induces cell cycle entry in FL5.12 that is suppressed by ACLY targeting. Impairment of *de novo* lipid synthesis does play a significant role in slowing cell replication [110], but ACLY downregulation also promotes cell differentiation, indicating that enhanced acetyl-CoA abundance can be critical for cell identity and possibly for tumorigenesis [110]. More recent studies confirmed that ACLY-dependent histone acetylation contributes to the maintenance of stemness and to the regeneration of the haematopoietic compartment after chemotoxicity [111].

It is now well appreciated that pluripotency is maintained through the tight control of metabolite availability, as discussed above. However, more work is required to understand the contribution of this metabolic-epigenetic signalling axis to haematopoietic cell renewal in homeostasis and after injury.

### 6.2. Activated B cells

Cell response to pathogen encounter must be rapid and effective and requires a massive change in cellular function. To this goal, activated B cells rapidly re-shape their metabolism and their nuclear architecture [112]. Interestingly, *in vitro* stimulation with LPS of isolated naive B cells (which typically induce extra-follicular effector cells) induces acute accumulation of acetyl-CoA within 12 hours; in turn, this leads to ACLY-dependent global hyperacetylation of the epigenome and a boost in transcriptional activity [112]. Similarly, methylation-impacting TCA intermediates (a-KG, succinate, fumarate) are rapidly accumulated upon initial B cell activation [74]. This is consistent with genome-wide removal of repressive H3K27me3 and H3K9me3 for chromatin decondensation observed in activated B cells [113].

Metabolic fluctuations are extremely quick and readily respond to extracellular stimulations [114]. The innate urgency of B cell response makes metabolites ideal signalling mediators; their ability to integrate extracellular sensing into the epigenome is certainly exploited by B cells for activation. This is particularly clear during the germinal centre reaction (expansion, SHM/CSR and block of differentiation) and during plasma cell maturation (terminal differentiation and antibody production).

### 6.3. Germinal centre (GC) B cells

Within GC, activated B cells improve their affinity for a cognate antigen through iterative rounds of somatic hypermutations (SHM) of their variable light chain Ig genes. Expression of *Aicda* (AID), which catalyses SHM, is a hallmark of GC B cells and is controlled by epigenetic regulation [55]; interestingly, multiple metabolic mechanisms contribute to defining the epigenetic profile of the *Aicda* locus indicating that changes in the metabolic activity are critical for the control of gene expression during B cell clonal selection and expansion.

Murine B cells stimulated with LPS and IL-4 exhibit reduced NAD+/NADH ratio [55], due to increased glycolysis [73,75] as NAD is reduced throughout this pathway. In glucose-repleted conditions, the activity of NAD-dependent de-acetylases like SIRT1 is also reduced, which has a direct impact on the acetylation of histones (H3K9ac, H3K14ac - elevated) at the *Aicda* promoter [55]. Reduced SIRT1 activity also leads to hyper-acetylation of *DNA-methyltransferase 1* (DNMT1), which decreases both its catalytic activity and its recruitment to *Aicda* promoter. Together this indicates that decreased deacetylation of histone and non-histone proteins upon B cell activation boosts transcription of *Aicda* gene, promotes class-switch recombination and clonal expansion of activated B cells in germinal centres.

The intake of dietary fibres is associated with the mounting of an efficient humoral response [115]. Interestingly, conserved *Aicda* regulatory regions have been reported to be highly H3-acetylated in response to dietary fibres ingestion [116]. This is linked to the increased systemic availability of short-chain fatty acids (SCFAs, *see below*) generated by the gut microbiome from the fermentation of dietary fibres; SCFAs increase cellular acetyl-CoA levels in isolated CD40-stimulated B cells [116]. This shows that both signalling and environmental cues drive fluctuations of metabolites that influence the epigenetic signature at sites that are critical for GC development.

**Figure 5.**
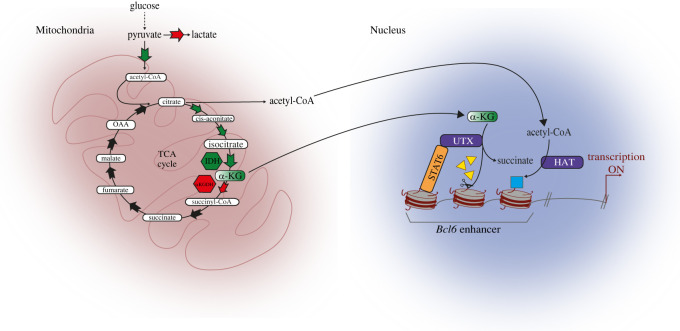
Metabolic control of epigenetic reprogramming in the germinal centre reaction. Findings from Haniuda and coworkers [57] using an *in vitro* model of murine GC reaction. Upon CD40/BAFF stimulation, mature B cells rewire their mitochondrial metabolism. Elevated flux through the pyruvate dehydrogenase (PDH) converts glucose-derived pyruvate into TCA intermediates. Levels of alpha-ketoglutarate (a-KG) are significantly augmented, due also to upregulation of a-KG-generating enzyme isocitrate dehydrogenase (IDH) and downregulation of a-KG-processing enzyme a-KG dehydrogenase (aKGDH). Increased intracellular abundance of a-KG enhances activity of histone demethylase UTX, leading to ipo-methylation of the *Bcl6* promoter. Increased acetylation was also observed. Green bold arrows highlight reactions enhanced upon stimulation; in contrast, red bold arrows denote decreased flux.

The transcriptional profile of B cells undergoing the GC reaction is orchestrated by the master transcriptional repressor *B-Cell Lymphoma factor 6* (BCL6) that controls a number of cellular functions including activation, cytokine-signalling, DNA-damage response and cell cycle progression [117]. GC B cells show well-defined cellular features, most of which are overplayed during lymphomagenesis such as genetic instability (e.g. SHM), clonal proliferation, enhanced mobility, immune evasion and metabolic deregulation [38,39]. Acquisition of these traits is coordinated by BCL6, but cellular metabolism exerts a backward influence on BCL6 expression through locus-specific regulation of the epigenome. In GC dark zone, B cells show a markedly oxidative metabolism, with an elevated mitochondrial mass and maximized mitochondrial respiration for energy production despite conducting minimal glycolysis and rely on fatty acid oxidation (FAO) to replenish the TCA cycle [76]. It is worth noting that high rates of oxidative phosphorylation are uncommon in fast proliferating cells that tend to prioritize glycolysis and the feeding of branching pathways for the generation of intermediates useful for cell division such as nucleotides and glycolipids [66]. By contrast, GC B cells accumulate TCA cycle intermediates, most notably alpha-ketoglutarate (α-KG). *In vitro* modelling of GC reaction—where primary cells are stimulated with CD40 L, BAFF and IL4—shows elevation of α-KG and reduction of histone methylation both at the global level and at the *Bcl6* locus [57]. The loss of epigenetic suppressive marks contributes to the upregulation of *Bcl6*, linking metabolic reprogramming, epigenetic state and cell differentiation [57]. Interestingly, higher levels of acetylated H3K27 were observed under the same conditions, consistent with elevation of acetyl-CoA abundance [76]. In summary, oxidative GC B cells exploit accumulation TCA intermediates to regulate the epigenetic status of *Bcl6* enhancer contributing to its tightly controlled expression (figure 5).

### 6.4. Plasma cells

Selection for high-affinity BCR leads to the generation of long-lived plasma cells or memory B cells, together known as effector B cells. The production and secretion of antibodies represent distinct metabolic challenges that need to be met shortly after effector cells re-encounter the antigen. LPS-stimulated B cells augment acetyl-CoA availability promptly after stimulation by increasing carbon flux to mitochondria and enhancing the export of citrate [74,118]. The consequence is a marked increase in both global levels of histone acetylation and lipid synthesis: the former facilitates a boost in transcription while the latter is necessary to enlarge the ER compartment that sustains the secretory activity [112,118]. Together these studies highlight a two-pronged role of acetyl-CoA in plasma cell specification: supporting biomass generation and mediating epigenetic rearrangements.

Increased expression of ACLY upon LPS stimulation has been reported and might contribute to elevation of acetyl-CoA levels [118]. The rapidity of acetyl-CoA accumulation however suggests that altered nutrient uptake and utilization might be more predominant contributors. Catabolism of poly-unsaturated fatty acids (PUFA) has been shown to be critical for plasma cell differentiation and antibody production [119]. Indeed, mice deficient for FABP3, a long-chain fatty acids carrier that preferentially binds PUFAs, show decreased BLIMP-1 levels due to reduced H3K27 acetylation of *Prdm-1* promoter [119]. In line, plasma cell development and IgM production are impaired in FABP3-deficient mice, although the link between FABP3 activity and epigenetic regulation of B cell development is unclear. FABP3 is not known to interact with chromatin writers; it is fair to speculate that FABP3 activity impacts histone acetylation by modulating intracellular acetyl-CoA levels. It is tempting to wonder whether alterations of PUFA dietary intake modify acetyl-CoA availability in activated B cells and test their effect on epigenetic reprogramming and antibody production.

Short-chain fatty acids (SCFAs) are fatty acids with fewer than six carbon atoms. They originate from a wide spectrum of biochemical reactions but in mammals they are mostly produced by intestinal microbes. The most abundant are acetate (C2, CH_3_COOH), propionate (C3, CH_3_CH_2_COOH) and butyrate (C4, CH_3_(CH_2_)_2_COOH). SCFAs levels rely on dietary fibre and alcohol intake, and fluctuate significantly across different organs and during the day, while playing diverse physiological functions [120]. SCFAs availability impacts the epigenome in multiple ways. First, they can be used as anaplerotic source to feed the TCA cycle and contribute to the pool of epigenome-influencing intermediates (e.g. acetyl-CoA). Similarly, acetate can also be directly converted to acetyl-CoA by ACSS2, a mechanism that mediates systemic effects and impacts histone acetylation and lipid synthesis in cancer and several other conditions [121–123]. In addition, (iso)butyrate is a wide-spectrum competitive inhibitor of histone acetyl transferases (HATs) and high levels drive histone hyperacetylation in the colonic epithelium [124,125]. Lastly, propionate can be converted to propionyl-CoA, which has recently been found to be highly enriched in the nucleus where it is used for histone propionylation [114]. Together with the fact that SCFAs levels fluctuate significantly in the circulation, this hints at the possibility that SCFAs might mediate dietary influence on humoral immunity [116]. Indeed, dietary fibres intake impacts the number of IgA-positive (IgA+) plasma cells in the systemic circulation and in the gut, although divergent effects have been reported in experimental models [116,126] likely due to dysbiosis and dose-dependent effects. When administered to immunocompetent mice, SCFAs induce both decrease [126] and increase [116] of plasma cell count and antibody production both *in vitro* and *in vivo*. However, SCFAs administration consistently increase histone H3 acetylation at regulatory regions [116,126]. While the impact of dietary fibres on B cell effector function is still elusive, it is likely that SCFAs derived from the fermentation of dietary fibres cause epigenetic reprogramming of plasma B cells both at the intestinal and at the systemic level with consequences on the humoral response.

A lesser-abundant SCFA is panthoneate (C5, vitamin B5), which is the precursor of coenzyme A (CoA) and is also either generated by the gut microbiome or ingested. It has been reported that panthoneate administration in mice ameliorates experimental inflammation by feeding acetyl-CoA synthesis and boosting histone acetylation in multiple immune cells including regulatory B cells, a small population of immune-modulating B cells [127].

Interestingly, stimulation of human CD19+ B cells with TLR-9, BCR and IFN-α induces plasmablast differentiation together with an increase in the uptake of methionine, while methionine deficiency suppresses the expression of key genes for plasmablast differentiation (*Bach2, Prdm1* and *Xbp1*) and causes a decrease in PC count. Interestingly, both DNA and histone methylation at the *Bach2* locus are impacted by methionine availability, suggesting that methionine-derived SAM could be regulated during cell specification to support epigenetic remodelling [128]. Micronutrient intake has also been shown to impact DNA methylation, plasma cell differentiation and antibody production both *in vitro* and *in vivo* [129]. Ascorbate (vitamin C) deficiency has been long associated with increased susceptibility to infections [130] and interestingly it is a critical cofactor for the only known class of DNA demethylating enzymes, TET-1/2/3, which oxidize 5-methylcytosine into 5 hydroxymethylcytosine (5hmC) [129]. Ascorbate supplementation alters DNA methylation at several promoters, including *Prdm1* (which encodes BLIMP1), and impairs STAT3 binding in IL21-stimulated B cells [131]. As mentioned above, iron (Fe2+) availability is critical for the activity of 2-oxoglutarate-dependent dioxygenases that remove methyl moieties from methylated histone residues. Iron deficiency determines profound epigenetic disturbances in antibody-producing B cells (both plasma cells [132] and memory B cells [133]) that affect their activation and, ultimately, the humoral response.

Collectively, these findings highlight a relationship between cellular metabolism and epigenetics, both being extensively reprogrammed during B cell specification. While epigenetic factors primarily guide the reshaping of cellular identity and function across different steps of maturation, metabolic intermediates integrate heterogeneous signals into the epigenome to inform about the fitness of extracellular conditions and provide an additional level of control against undesired adaptative responses.

## 7. Metabolic-epigenetic interplay in B cell lymphomagenesis

Lymphomas are a heterogeneous group of malignancies that originate from either haematopoietic precursors or different developmental stages of B- and T-lymphocytes. For B cells, the initiation of lymphomagenesis can often be tracked back to the set of events that constitute the GC reaction [5,38]; indeed, many features that typify GC B cells (i.e. genomic instability, clonal expansion, block of differentiation, and others [38]) are hijacked and amplified by oncogenic signals. In line, non GC-derived lymphomas are less frequent and usually more indolent. Main feats of B cell lymphomagenesis are summarized in box 2.

Box 2. **Lymphomagenesis of B-cell non-Hodgkin lymphomas.**

Non-Hodgkin lymphomas (NHLs) constitute a highly heterogeneous group of malignant neoplasms arising from lymphoid tissues, in large part from transient anatomical sites that host the germinal centre (GC) reaction. Indeed, traits that typify GC B cells (i.e. genomic instability, a highly proliferative phenotype, metabolic plasticity, block of terminal differentiation [35] and immune evasion [38]) are also hallmarks of cancer cells.

According to 2016 WHO classification, B-NHLs comprise: Burkitt lymphoma (BL), which derives from dark-zone GC B cells; follicular lymphoma (FL) and germinal centre B cell-like diffuse large B cell lymphoma (GCB-DLBCL), deriving from light zone GC B cells; and activated B cell-like diffuse large B cell lymphoma (ABC-DLBCL), which arises from post-GC B cells committing to plasmablast differentiation.

Metabolic, epigenetic and signalling programmes coordinating the GC reaction are hijacked by oncogenic lesions, including chromosomal translocations, amplifications, deletions, point mutations, and aberrant somatic hypermutation and drive B-cell lymphomagenesis.

**Burkitt lymphoma**

BL is a highly aggressive B-NHL affecting children and young adults. It is characterized by *MYC* translocation to the immunoglobulin loci, usually generated by dysfunctional activity of the DNA-modifying enzyme Activation-Induced cytidine Deaminase (AID). This results in deregulation of *MYC* proto-oncogene in BL B cells that sustains uncontrolled cell proliferation and growth and further induces genomic instability. Under physiological conditions, *MYC* expression is turned on early during GC formation and is then repressed by BCL6 when cells move to the dark zone. Beside *MYC* overexpression, 70% BL patients can bear mutations in *Tcfe* which result in defective E2A and uncontrolled BCR signalling. Even tumour-suppressor genes like TP53 and PTEN are often mutated in BL patients.

**Follicular lymphoma**

FL is an indolent B-NHL which may evolve into more aggressive forms. Tumorigenesis of FL is widely recognized to be a consequence of BCL2 ectopic expression after t(14;18) translocation and its evasion from BCL6-mediated repression. IgH-Bcl2 t(14;18) translocation is caused by defective RAG-mediated V(D)J recombination during early B cell maturation in the bone marrow. Nevertheless, most FL patients also carry mutations in chromatin modifying enzymes such as CREBBP/EP300, KMT2D and EZH2.

**Diffuse large B cell lymphoma**

DLBCL is the most common B-cell lymphoma and is characterized by remarkable genetic and epigenetic heterogeneity. Interestingly, more than 50% of genes mutated in DLBCL are chromatin modifiers. Common mutations are loss-of-function of CREBBP/EP300 and KMT2D in all DLBCL subtypes and gain-of-function of EZH2 in.the GCB-subtype. Importantly, *in vitro* and *in vivo* studies confirmed that epigenetic rearrangements *per se* drive DLBCL lymphomagenesis by inducing changes in gene expression that overplay some feature of GC B cells. In addition, histone proteins can be themselves mutated; genetic ablation of linker histone H1, which is often lost in DLBCL, initiates malignant transformation *in vitro* [134].

KMT2D induces H3K4 mono-methylation at active enhancers that controls B cell fate decision. It is the most commonly mutated gene in DLBCL, in ∽30% of all cases. In vivo studies have revealed that its loss coupled to Bcl2 deregulation promotes lymphomagenesis [15]. Upon Kmdt2d mutation, downregulation of genes involved in immune signalling (IL, TNF, CD40) and plasma cell differentiation has been observed in both human and mice [135].

Acetyltransferases CREBBP and EP300 are mutated in 25% and 5% of DLBCL cases, respectively. Their mutation is predominantly observed in GCB-DLBCL subtype [136]. Crebbp deletion coupled to myc ectopic expression in mice leads to earlier lymphoma development in mice, while xenografts from mutant CREBBP human DLBCL cells expand faster and to a greater extent than tumours of wild-type CREBBP human DLBCL [137].

Mutation of EZH2 at Y641, which is observed in more than 20% DLBCL cases, increases H3K27 trimethylation and represses expression of antiproliferative and tumour suppressor genes such as Cdkn1a and Prdm1 [136]. In mice, mutant EZH2 induce a hyper-proliferative state of GC B cells culminating in GC hyperplasia. However, only in combination with deregulated BCL2 can mutant EZH2 induce DLBCL [13].

The pivotal role of epigenetics in B cell development aligns well with the notion that B cell malignancies, especially lymphomas, have a strong epigenetic basis [138,139]. Aberrant DNA methylation, histone modifications profile and even chromatin architecture are staples of B cell malignancies, and inhibitors of DNA/histone methyltransferases are tested for the treatment of B cell lymphomas [140,141]. Chromatin writers and erasers are frequently mutated in GC-derived lymphomas, mostly in diffuse large B cell lymphomas (DLBCLs) that show prevalent mutations in major epigenetic modifiers such as EZH2, EP300, CREBBP and KMT2D [139] (box 2).

At the same time, deregulation of cellular metabolism is a hallmark of cancer; in fact, haematological malignancies are characterized by numerous metabolic disturbances, which in part contribute to the epigenetic aberrations that promote lymphomagenesis [85]. We will review here emerging evidence documenting metabolic-epigenetic crosstalk in B cell lymphomas with the reasoning that metabolic vulnerabilities might potentiate epigenetic-based therapies that are impacted by intratumoral heterogeneity.

### 7.1. Metabolic-dependent histone and DNA methylation in B cell lymphomas

Both DNA and histone methylation are sensitive to metabolic reprogramming [31]; converging evidence shows that rewiring of one-carbon metabolism or changes in α-KG availability play a significant role in B cell malignancies.

Burkitt lymphoma (BL) is a rapid-growing B cell neoplasia caused by *c-myc* dysregulation mostly due to chromosomal translocations [142]. Interestingly, *c-myc* regulates the expression of PHGDH and PSAT1, two rate-limiting enzymes in *de novo* serine biosynthesis that are also upregulated in BL patients [143]. This feeds one-carbon metabolism and sustains S-adenosyl methionine (SAM) levels. Indeed, inhibition of either PHGDH or PSAT1 leads to a significant reduction in global levels of histone H3K27 tri-methylation and proliferation arrest [143]. In summary, MYC augments SAM abundance inside the cell feeding serine biosynthesis and one-carbon metabolism.

The finding that the *Serine Hydroxymethyltransferase 2* (SHMT2) is an oncogenic driver of BCL2-expressing lymphomas [144] further underscores the crucial role of serine one-carbon metabolism in B cell pathology. SHMT2 is a mitochondrial enzyme that catalyses the reversible reaction of serine and tetrahydrofolate (THF) to glycine and 5,10-methylene THF and participates in the shuttling of methyl carbon units between the cytoplasm and mitochondria [145]. *SHMT2* is overexpressed in follicular lymphomas (FLs) and DLBCLs, the two most frequent forms of B-cell non-Hodgkin lymphomas (NHLs) [144]. Amplification of SHMT2 is sufficient to drive lymphomagenesis in autochthonous mouse models of FL (*VavP-Bcl2*, where the antiapoptotic gene BCL2 is expressed in multiple haematopoietic lineages) by sustaining SAM generation. Increased SAM availability leads to a hypermethylated phenotype that promotes the expression of pro-lymphomagenic genes [144].

Together these data indicate that rewiring of activated methyl units is important for the reprogramming of DNA and histone methylation in multiple forms of NHL. At the same time, genetic alterations that impact α-KG levels have been described in DLBCLs, the most common type of B-cell NHL [138]. In a small set of DLBCLs, *D-2-hydroxyglutarate dehydrogenase* (D2HGDH) haploinsufficiency results in significantly decreased α-KG levels [146]. D2HGDH is a FAD-dependent mitochondrial enzymes that converts 2-hydroxyglutarate (2-HG), a useless and eventually malignant cellular metabolic byproduct, into α-KG; hence, the physiological role of D2HGDH is to prevent the deleterious accumulation of 2-HG produced under conditions of cellular stress [146,147]. In DLBCL tumours, D2HGDH deficiency (or inactivating mutations) lowers α-KG levels while augmenting both 2-HG abundance and DNA/histone methylation [146]. The near complete absence of D2HGDH mutations in normal samples points to the conclusion that constraining 2-HG production is a critical gatekeeper of B cell transformation, which is consistent with the finding that D2HGDH is transcriptionally upregulated by oncogenic MYC [148].

On the other hand, inhibitors of the histone methyl transferase EZH2 show some efficacy for the treatment of DLBCL but resistance eventually occurs [141]. Sensitivity to EZH2 inhibition is determined by fluctuations of methionine catabolism [128], which feeds *de novo* SAM generation. In principle, availability of the universal methyl donor SAM influences the response to suppression of histone methylation, highlighting the crucial (yet elusive) role of cell metabolism in DLBCL refractoriness to therapy.

In conclusion, metabolic cofactors (SAM, α-KG) are instrumental for the reprogramming of methylation status in B cell tumorigenesis and their cellular availability is often perturbed by oncogenic mutations and signalling.

### 7.2. Regulation of histone deacetylases by endogenous metabolites

Histone acetylation is widely deregulated in haematological disorders. This has been linked to the high prevalence of mutations at genes encoding either histone acetylatransferases (HATs) or histone deacetylases (HDACs) [38]. HDAC inhibitors have been tested for the treatment of multiple diseases, although their effects and mechanisms of action are variable across cancer types. As yet, they have been approved for the treatment of multiple myeloma and T-cell lymphomas [149], but for the most part they are used only in combination with other anti-neoplastic drugs. On the other hand, they display lower efficiency in B cell lymphomas [62,150]. Most B tumour cell lines show a hyperacetylated epigenome [56]; while this confers superior sensitivity to bromodomain inhibition (e.g. JQ1) [56], it might reduce the cytotoxic effects of deacetylase inhibitors. It also suggests that GC-derived lymphomas might rely on high levels of histone acetylation for the expression of pro-tumorigenic genes or activation of cancer-promoting super-enhancers (SE) [151].

However, the inefficiency of HDAC inhibitors might be explained also by metabolic influence. Indeed, DLBCL cell lines exhibit heterogeneous response (i.e. xenograft tumour growth) to HDAC inhibitors, and high OXPHOS activity correlates with poor response [152]. In addition, the FDA-approved HDAC inhibitor panobinostat, induces rapid metabolic reprograming in DLBCL tumour cells with marked changes in choline pathway metabolites for the synthesis of membrane phospholipids [62]. Similarly, betaine plasma levels of relapsed/refractory DLBCL patients decrease significantly after the first cycle of panobinostat treatment, suggesting that DLBCL cells develop choline dependency upon panobinostat administration [62]. Several questions remain open: which is the mechanistic link between phospholipid biosynthesis and HDAC inhibitor resistance? And how do transformed B cells rewire choline metabolism? Regardless of our poor understanding of the relationship between HDAC inhibition and metabolic remodelling, these data clearly indicate that specific pathways of B cell metabolism are linked to protein deacetylation.

While canonical HDAC inhibitors are ineffective, SIRT3 inhibitor YC8–02 alone showed remarkable cytotoxicity for DLBCL cells both *in vivo* and *in vitro*; at the same time, genetic targeting of *Sirt3* reduced proliferation of DLBCL cell lines and tumour growth in spontaneous mouse models of MYC-induced lymphomagenesis [153]. Notably, both treatment with YC8-02 and *Sirt3* knockout have little effect on the physiological development of B cells [153]. Elevated SIRT3 activity maintains high levels of TCA cycle intermediates, including acetyl-CoA and α-KG, through anaplerotic glutaminolysis. This explains the counterintuitive finding that SIRT3 sustains H3 acetylation and unveils a critical metabolic-epigenetic vulnerability of DLBCL cells [153].

In conclusion, the evidence collected so far reveals that histone deacetylation significantly impacts the DLBCL metabolic network. However, the mechanisms remain largely elusive, and a more direct contribution of intracellular and/or extracellular metabolites to histone acetylation in lymphomagenesis has quite surprisingly not been investigated.

## 8. Conclusion

The epigenetic blueprint of the cell determines its identity and function [154]. Progression through different developmental stages depends on the dynamic remodelling of chromatin landscape, which is carried over by large ATP-dependent complexes that alter the modular structure of the chromatin or by enzymes that can epigenetically modify DNA and histone proteins. The latter play a critical role in lymphocytes specification and transformation, which has led to increasing interest in the application of epigenetic-based drugs to immunological disorders.

The relatively limited success of ‘epidrugs’ hints on the fact that non-enzymatic factors participate in epigenetic remodelling. Indeed, metabolites are essential cofactors or substrates for chromatin-modifying enzymes. Their levels are in dynamic equilibrium with a number of systemic, microenvironmental and cell-intrinsic cues, hence oscillations inform cell fate decisions [24,26]. Compelling evidence demonstrates that metabolic inputs significantly contribute to the epigenetic state of the cell and, in doing so, they exert control over cellular reprogramming during differentiation [24,28,155].

Here we provided context for the notion that metabolic rewiring contributes to the epigenomic changes that guide the evolution from naive B cell precursors to germinal centre-reacting cells to antibody-secreting cells. The metabolic setup sustains biosynthetic demands in a stage-specific manner but also cooperate to define the epigenetic code of the cell. Interestingly, the link between metabolic and epigenetic reprogramming is extensively characterized in T lymphocytes, where evidence shows that this axis is involved in the CD4/CD8 switch [24], the development of effector cells [90], the formation of regulatory T cells [156,157] while also being hijacked in cancer to drive T cell exhaustion [158,159]. Despite the studies summarized here, modalities of metabolism/epigenetics connection in B cell biology remain much more elusive.

The superior genomic and epigenomic complexity of B cell pathophysiology and the lack of well-characterized *in vitro* models (e.g. for GC reaction) explain in part the disparity. Yet, this topic deserves more indepth investigation. The success story of ivosidenib and enasidenib (mutant IDHs inhibitors) [160] clearly underscores the translational implications that lay in targeting the metabolic-epigenetic axis. In addition, metabolism is easily modifiable using dietary or pharmacological interventions; diet or well-characterized metabolic drugs (i.e. statins) might synergize with available epidrugs. For example, choline kinase inhibitors have been shown to overcome resistance in DLBCL tumours treated with the HDAC inhibitor panobinostat [62]. The proof-of-concept for this framework is provided by the finding that histidine-restricted diet sensitizes leukaemic cells to methotrexate [161]; while evidence is mounting for solid cancers, more research is vital for haematological malignancies [63].

It is finally noteworthy that, while metabolic intermediates exert a significant control over the epigenome, there is a considerable reciprocal influence between metabolic and epigenetic reprogramming. In fact, chromatin rearrangements often coordinate changes in metabolic fluxes by regulating gene expression [69] or buffering metabolite levels [162].

All in all, the metabolic–epigenetic connection is an important signalling axis in lymphocyte biology that has been only sporadically investigated in the context of B cell pathophysiology. However, results strongly support its key role in B cell maturation and malignant transformation.

## Data Availability

This article has no additional data.
